# The emergence of a field: a network analysis of research on peer review

**DOI:** 10.1007/s11192-017-2522-8

**Published:** 2017-10-03

**Authors:** Vladimir Batagelj, Anuška Ferligoj, Flaminio Squazzoni

**Affiliations:** 1 Department of Theoretical Computer Science, Institute of Mathematics, Physics and Mechanics, Jadranska 19, 1000 Ljubljana, Slovenia; 2 Andrej Marušič Institute, University of Primorska, Muzejski trg 2, 6000 Koper, Slovenia; 3 Faculty of Social Sciences, University of Ljubljana, Kardeljeva pl. 5, 1000 Ljubljana, Slovenia; 4 Department of Economics and Management, University of Brescia, Via San Faustino 74/B, 25122 Brescia, Italy

**Keywords:** Peer review, Journals, Authors, Citation networks, Main path

## Abstract

This article provides a quantitative analysis of peer review as an emerging field of research by revealing patterns and connections between authors, fields and journals from 1950 to 2016. By collecting all available sources from Web of Science, we built a dataset that included approximately 23,000 indexed records and reconstructed collaboration and citation networks over time. This allowed us to trace the emergence and evolution of this field of research by identifying relevant authors, publications and journals and revealing important development stages. Results showed that while the term “peer review” itself was relatively unknown before 1970 (“referee” was more frequently used), publications on peer review significantly grew especially after 1990. We found that the field was marked by three development stages: (1) before 1982, in which most influential studies were made by social scientists; (2) from 1983 to 2002, in which research was dominated by biomedical journals, and (3) from 2003 to 2016, in which specialised journals on science studies, such as Scientometrics, gained momentum frequently publishing research on peer review and so becoming the most influential outlets. The evolution of citation networks revealed a body of 47 publications that form the main path of the field, i.e., cited sources in all the most influential publications. They could be viewed as the main corpus of knowledge for any newcomer in the field.

## Introduction

Peer review is key to ensure rigour and quality of scholarly publications, establish standards that differentiate scientific discoveries from other forms of knowledge and maintain credibility of research inside and outside the scientific community (Bornmann 2011). Although many believe it has roots that trace back centuries ago, historical analysis indicated that the very idea and practices of peer review that are predominant today in scholarly journals are recent. Indeed, peer review developed in the post-World War II decades when the tremendous expansion of science took place and the “publish or perish” culture and their competitive symbolisms we all know definitively gained momentum (Fyfe et al. 2017). Unfortunately, although this mechanism determines resource allocation, scientist reputation and academic careers (Squazzoni et al. 2013), a large-scale quantitative analysis of the emergence of peer review as a field of research that could reveal patterns, connections and identify milestones and developments is missing (Squazzoni and Takács 2011).

This paper aims to fill this gap by providing a quantitative analysis of peer review as an emerging field of research that reveals patterns and connections between authors, fields and journals from 1950 to 2016. We collected all available sources from Web of Science (WoS) by searching for all records including “peer review” among their keywords. By using the program **WoS2Pajek** (Batagelj 2007), we transformed these data in a collection of networks to reconstruct citation networks and different two-mode networks, including works by authors, works by keywords and works by journals. This permitted us to trace the most important stages in the evolution of the field. Furthermore, by performing a ’main path’ analysis, we tried to identify the most relevant body of knowledge that this field developed over time.

Our effort has a twofold purpose. First, it aims to reconstruct the field by quantitatively tracking the formation and evolution of the community of experts who studied peer review. Secondly, it aims to reveal the most important contributions and their connections in terms of citations and knowledge flow, so as to provide important resources for all newcomers in the field. By recognizing the characteristics and boundaries of the field, we aim to inspire further research on this important institution, which is always under the spotlight and under attempts of reforms, often without relying on robust evidence (Edwards and Roy 2016; Squazzoni et al. 2017).

For standard theoretical notions on networks we use the terminology and definitions from Batagelj et al. (2014). All network analyses were performed using **Pajek**—a program for analysis and visualization of large networks (De Nooy et al. 2011).

## Data

### Data collection

**Fig. 1:**
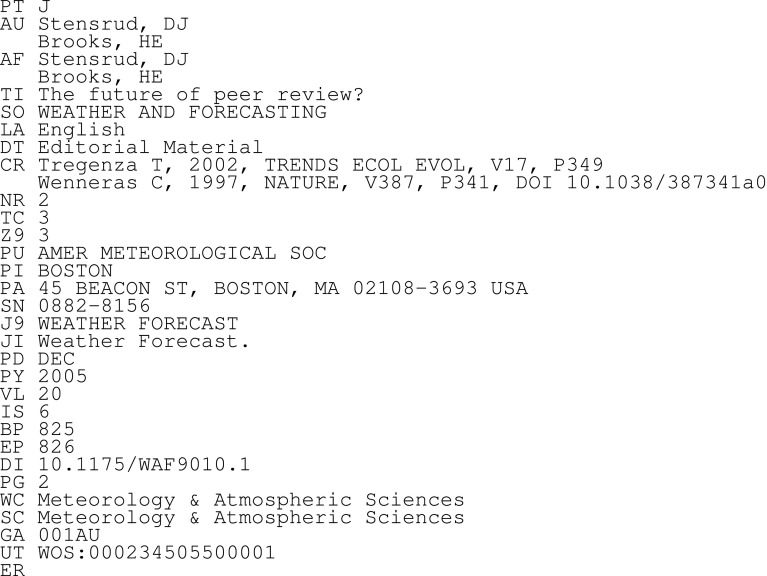
Record from web of science

We searched for any record containing “peer review*” in WoS, Clarivate analytics’s multidisciplinary databases of bibliographic information in May and June 2015. We obtained 17,053 hits and additional 2867 hits by searching for "refereeing". Figure 1 reports an example of records we extracted. We limited the search to the WoS core collection because for other WoS databases the CR-fields (containing citation information) could not be exported.

Using **WoS2Pajek** (Batagelj 2007), we transformed data in a collection of networks: the citation network ${\mathbf {Cite}}$ (from the field CR), the authorship network ${\mathbf {WA}}$ (from the field AU), the journalship network ${\mathbf {WJ}}$ (from the field CR or J9), and the keywordship network ${\mathbf {WK}}$ (from the field ID or DE or TI). An important property of all these networks is that they share the same set—the set of works (papers, reports, books, etc.) as the first node set *W*. It is important to note that a citation network ${\mathbf {Cite}}$ is based on the citing relation ${\mathbf {Ci}}$

$$\begin{aligned} w\,{\mathbf {Ci}}\, z \equiv {\text{ work }}\, w \,{\text{ cites } \text{ work }}\, z \end{aligned}$$

Works that appear in descriptions were of two types:

- Hits—works with a WoS description;
- Only cited works (listed in CR fields, but not contained in the hits).

These data were stored in a partition *DC*: ${ DC}[w]=1$ iff a work *w* had a WoS description; and ${ DC}[w]=0$ otherwise. Another partition *year* contained the work’s publication year from the field PY or CR. We also obtained a vector *NP*: $N P[w] =$ number of pages of each work *w*. We built a CSV file titles with basic data about works with ${ DC}=1$ to be used to list results. Details about the structure of names in constructed networks are provided in “The structure of names in constructed networks” section.

The dataset was updated in March 2016 by adding hits for the years 2015 and 2016. We manually prepared short descriptions of the most cited works (fields: AU, PU, TI, PY, PG, KW; but without CR data) and assigned them the value ${ DC}=2$.

A first preliminary analysis performed in 2015 revealed that many works without a WoS description had large indegrees in the citation network. We manually searched for each of them (with indegree larger or equal to 20) and, when possible, we added them into the data set. It is important to note that earlier papers, which had a significant influence in the literature, did not often use the now established terminology (e.g., keywords) and were therefore overlooked by our queries.

After some iterations, we finally constructed the data set used in this paper. The final run of the program **WoS2Pajek** produced networks with sets of the following sizes: works $|W| = 721{,}547$, authors $|A| = 295{,}849$, journals $|J| = 39{,}988$, and keywords $|K| = 36{,}279$. In both phases, 22,981 records were collected. There were 887 duplicates (considered only once).

We removed multiple links and loops (resulting from homonyms) from the networks. The cleaned citation network ${\mathbf {CiteAll}}$ had $n =721{,}547$ nodes and $m = 869{,}821$ arcs.

**Fig. 2:**
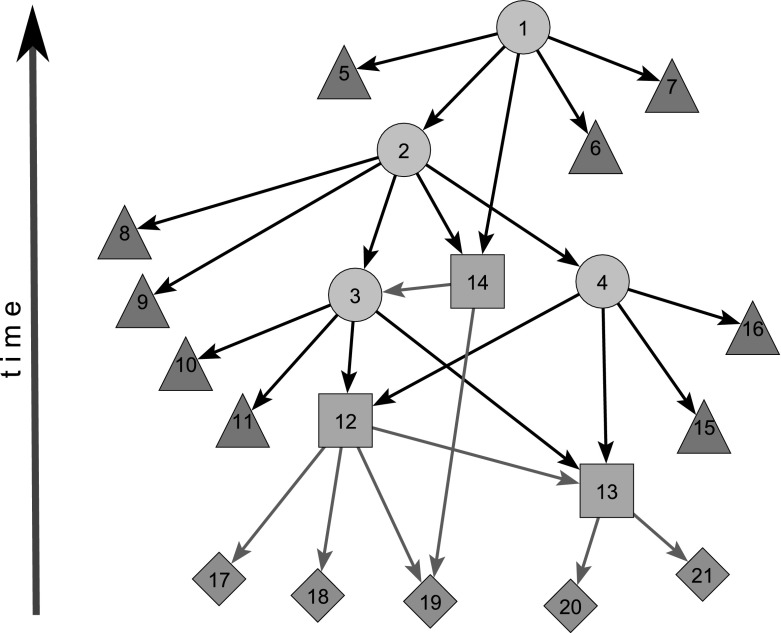
Citation network structure: ${ DC}=0$—circle, square; ${ DC}=1$—triangle, diamond

Figure 2 shows a schematic structure of a citation network. The circular nodes correspond to the query hits. The works cited in hits are presented with the triangular nodes. Some of them are in the following phase (search for often cited works) converted into the squares (found in WoS by our secondary search). They introduce new cited nodes represented as diamonds. It is important to note that the age of a work was determined by its publication year. In a citation network, in order to get a cycle, an “older” node had to cite a “younger or the same age” work. Given that this rarely happens, citation networks are usually (almost) acyclic.

To acyclic network’s nodes, we can assign levels such that for each arc, the level of its initial node is higher than the level of its terminal node. In an acyclic citation network, an example of a level is the publication date of a work. Therefore, acyclic networks can be visualized by levels—vertical axis representing the level with all arcs pointing in the same direction—in Fig. 2 pointing down.

In the following section, we look at some statistical properties of obtained networks.

## Distributions

**Fig. 3:**
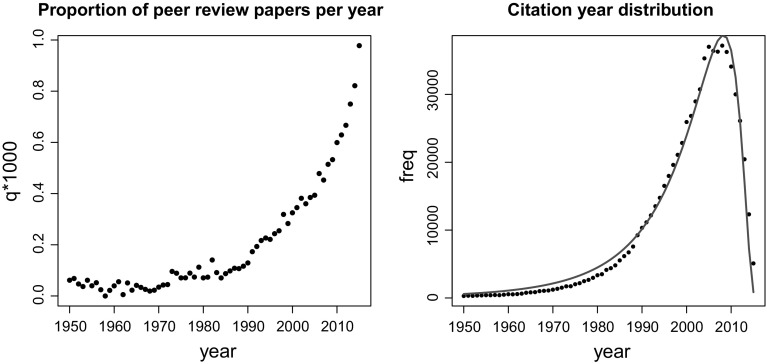
Growth of the number of works and the citation year distribution

In the left panel of Fig. 3, we showed a growth of the proportion *q*—the number of papers on peer review divided by the total number of papers from WoS (${ DC}>0$) by year. Proportions were multiplied by 1000. This means that peer review received growing interest in the literature, especially after 1990. For instance, in 1950 WoS listed only 6 works on peer review among 97,529 registered works published in that year, $q_{1950} = 0.6152 \times 10^{-4}$. In 2015, we found 2583 works on peer review among 2,641,418 registered works, $q_{2015} = 0.9779 \times 10^{-3}$.

In the right panel of Fig. 3, the distribution of all (hits $+$ only cited) works by year is shown. It is interesting to note that this distribution can be fitted by log normal distribution (Batagelj et al. 2014, pp. 119–121):

$$\begin{aligned} {\text{ dlnorm }}(x,\mu ,\sigma ) = \frac{1}{\sqrt{2 \pi } \sigma x}\, e^{-\frac{(\ln x - \mu )^2}{ 2 \sigma ^2}} \end{aligned}$$

**Fig. 4:**
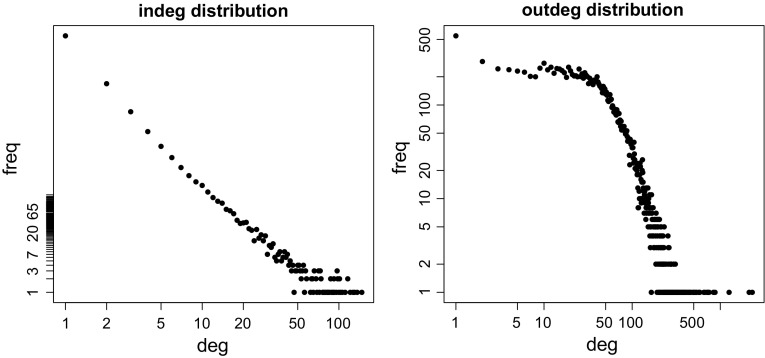
Degree distributions in the citation network

Figure 4 shows indegree and outdegree distributions in the citation network ${\mathbf {CiteAll}}$ in double logarithmic scales. Interestingly, indegrees show a scale-free property. It is somehow surprising that frequencies of outdegrees in the range [3, 42] show an almost constant value—they are in the range [215, 328]. works with the largest indegrees are the most cited papers.

**Table 1:** Most cited works

*n*	Freq	First author	Year	Title
1	173	Cohen, J	1988	Statistical power analysis for the behavioral sciences. Routledge
2	164	Peters, DP	1982	Peer-review practices of psychological journals—the fate of...Behav Brain Sci
3	151	Egger, M	1997	Bias in meta-analysis detected by a simple, graphical test. Brit Med J
4	150	Stroup, DF	2000	Meta-analysis of observational studies in epidemiology—a proposal for reporting. JAMA
5	135	Dersimonian, R	1986	Metaanalysis in clinical-trials. Control Clin Trials
6	130	Zuckerma, H	1971	Patterns of evaluation in science—institutionalisation, structure and functions of referee system. Minerva
7	130	Higgins, JPT	2011	Cochrane handbook for systematic reviews of interventions. Cochrane
8	126	Moher, D	2009	Preferred reporting items for systematic reviews and meta-analyses: the PRISMA statement. Plos Med
9	125	Higgins, JPT	2003	Measuring inconsistency in meta-analyses. Brit Med J
10	121	Cicchetti, DV	1991	The reliability of peer-review for manuscript and grant submissions...Behav Brain Sci
11	119	Hirsch, JE	2005	An index to quantify an individual’s scientific research output. Proc Natl Acad Sci Usa
12	114	Mahoney, M	1977	Publication prejudices: an experimental study of confirmatory bias...cognitive therapy and research
13	114	van Rooyen, S	1999	Effect of open peer review on quality of reviews and on reviewers’ recommendations:...Brit Med J
14	114	Easterbrook, PJ	1991	Publication bias in clinical research. Lancet
15	110	Landis, JR	1977	Measurement of observer agreement for categorical data. Biometrics
16	109	Godlee, F	1998	Effect on the quality of peer review of blinding reviewers and asking them to sign their reports—...JAMA
17	108	Horrobin, DF	1990	The philosophical basis of peer-review and the suppression of innovation. JAMA
18	107	Moher, D	2009	Preferred reporting items for systematic reviews and meta-analyses: PRISMA. Ann Intern Med
19	107	Jadad, AR	1996	Assessing the quality of reports of randomized clinical trials: Is blinding necessary? Control Clin Trials
20	105	Mcnutt, RA	1990	The effects of blinding on the quality of peer-review—a randomized trial. JAMA
21	104	Cole, S	1981	Chance and consensus in peer-review. Science
22	103	Moher, D	1999	Improving the quality of reports of meta-analyses of randomised controlled trials: QUOROM. Lancet
23	98	Justice, AC	1998	Does masking author identity improve peer review quality?—a randomized controlled trial. JAMA
24	97	Lock, S	1985	A difficult balance: editorial peer review in medicine. Nuffield Trust
25	95	van Rooyen, S	1998	Effect of blinding and unmasking on the quality of peer review—a randomized trial. JAMA
26	92	Black, N	1998	What makes a good reviewer and a good review for a general medical journal? JAMA
27	91	Scherer, RW	1994	Full publication of results initially presented in abstracts—a metaanalysis. JAMA
28	90	Higgins, JPT	2002	Quantifying heterogeneity in a meta-analysis. Stat Med
29	90	Smith, R	2006	Peer review: a flawed process at the heart of science and journals. J Roy Soc Med
30	87	Goodman, SN	1994	Manuscript quality before and after peer-review and editing at annals of internal-medicine. Ann Intern Med
31	87	Chubin, D	1990	Peerless science: peer review and US science policy. SUNY Press

Table 1 shows the 31 most cited works. Eight works, including the number 1, were cited for methodological reasons, not dealing with peer review. As expected, most of the top cited works were published earlier, with only eight published after 2000. We also searched for the most cited books. We found 15 books cited (number in parentheses) more than 50 times: (52) Kuhn, T: *The Structure of Scientific Revolutions*, 1962; (57) Glaser, BG, Strauss, AI: *The Discovery of Grounded Theory*, 1967; (67) Merton, RK: *The Sociology of Science*, 1973; (97) Lock, S: *A Difficult Balance*, 1985; (72) Hedges, LV, Olkin, I: *Statistical methods for meta-analysis*, 1985; (173) Cohen, J: *Statistical power analysis*, 1988; (87) Chubin, D, Hackett, EJ: *Peerless Science*, 1990; (60) Boyer, EL: *Scholarship reconsidered*, 1990; (51) Daniel, H-D: *Guardians of science*, 1993; (55) Miles, MB, Huberman, AM: *Qualitative data analysis*, 1994; (64) Gold, MR, et al.: *Cost-effectiveness in health and medicine*, 1996; (53) Lipsey, MW, Wilson, DB: *Practical meta-analysis*, 2001; (58) Weller, AC: *Editorial peer review*, 2001; (69) Higgins, JPT, Green, S: *Systematic reviews of interventions*, 2008; (130) Higgins, JPT, Green, S: *Systematic reviews of interventions*, 2011.

We also found that works having the largest outdegree (the most citing works) were usually overview papers. These papers have been mostly published recently (in the last ten years). Among the first 50 works that cited works on peer review most frequently, only two were published before 2000—one in 1998 and another one in 1990. However, none of them were on peer review and so we did not report them here.

### The boundary problem

Considering the indegree distribution in the citation network ${\mathbf {CiteAll}}$, we found that most works were referenced only once. Therefore, we decided to remove all ‘only cited’ nodes with indegree smaller than 3 (${ DC}=0$ and ${ indeg} < 3$)—the *boundary problem* (Batagelj et al. 2014). We also removed all only cited nodes starting with strings “[ANONYM”, “WORLD_”, “INSTITUT_”, “U_S”, “*US”, “WHO_”, “*WHO”, “WHO(”. “AMERICAN_”, “DEPARTME_”, “*DEP”, “NATIONAL_”, “UNITED_”, “CENTERS_”, “INTERNAT_”, “EUROPEAN_”. The final ‘bounded’ set of works $W_B$ included 45,917 works.

Restricting two-mode networks ${\mathbf {WA}}$, ${\mathbf {WJ}}$ and ${\mathbf {WK}}$ to the set $W_B$ and removing from their second sets nodes with indegree 0, we obtained *basic networks* ${\mathbf {WA}}_B$, ${\mathbf {WJ}}_B$ and ${\mathbf {WK}}_B$ with reduced sets with the following size $|A_B| = 62{,}106$, $|K_B| = 36{,}275$, $|J_B| = 6716$.

Unfortunately, some information (e.g., co-authors, keywords) was available only for works with a WoS full description. In these cases, we limited our analysis to the set of works with a description

$$\begin{aligned} W_D = \left\{ w \in W_B : DC[w] > 0 \right\} \end{aligned}$$

Its size was $|W_D| = 22{,}104$. By restricting basic networks to the set $W_D$, we obtained subnetworks ${\mathbf {WA}}_D$, ${\mathbf {WK}}_D$ and ${\mathbf {WJ}}_D$.

It is important to note that we obtain a *temporal network* ${\mathcal {N}}$ if the *time* ${\mathcal {T}}$ is attached to an ordinary network. ${\mathcal {T}}$ is a set of *time points* $t \in {\mathcal {T}}$. In a temporal network, nodes $v \in {\mathcal {V}}$ and links $l \in {\mathcal {L}}$ are not necessarily present or active in all time points. The node activity sets *T*(*v*) and link activity sets *T*(*l*) are usually described as a sequence of time intervals. If a link *l*(*u*, *v*) is active in a time point *t* then also its endnodes *u* and *v* should be active in the time point *t*. The time ${\mathcal {T}}$ is usually either a subset of integers, ${\mathcal {T}}\subseteq {\mathbb {Z}}$, or a subset of reals, ${\mathcal {T}}\subseteq {\mathbb {R}}$.

We denote a network consisting of links and nodes active in time, $t \in {\mathcal {T}}$, by ${\mathcal {N}}(t)$ and call it the (network) *time slice* or *footprint* of *t*. Let ${\mathcal {T}}' \subset {\mathcal {T}}$ (for example, a time interval). The notion of a time slice is extended to ${\mathcal {T}}'$ by: a time slice ${\mathcal {N}}({\mathcal {T}}')$ for ${\mathcal {T}}'$ is a network consisting of links and nodes of ${\mathcal {N}}$ active at some time point $t\in {\mathcal {T}}'$.

Here, we presented a simple analysis of changes of sets of main authors, main journals and main keywords through time (Tables 2, 3, 4, 5). Our analysis was based on temporal versions of subnetworks ${\mathbf {WA}}_D$, ${\mathbf {WK}}_D$ and ${\mathbf {WJ}}_D$—the activity times were determined by the publication year of the corresponding work.

Because of an increasing growth of interest (see the left panel of Fig. 3) on peer review, we decided to split the time line into intervals [1900, 1970], [1971, 1980], [1981, 1990], [1991, 2000], [2001, 2005], [2006, 2010], [2011, 2015].

## Most cited works, main works, journals and keywords

The left panel of Table 2 shows the authors with the largest number of co-authored works (${\mathbf {WA}}_{D}$ indegree), while the right panel shows the authors with the largest fractional contribution of works (weighted indegree in the normalized ${\mathbf {WA}}_{D}$). If we compare authors from Table 2 with the list of the most cited works in Table 1, we see that the two rankings are very different. Only three out of 25 authors with the largest number of works published a work that is on the list of 31 the most cited works. These are J. Cohen, D. Moher with two publications, and R. Smith. This is in line with the classic study by Cole and Cole (1973) in which they analyzed several aspects of the communication process in science. They used bibliometric data and survey data of the university physicists to study the conditions making for high visibility od scientist’s work. They found four determinants of visibility: the quality of work measured by citations, the honorific awards received for their work, the prestige of their departments and specialty. In short, quantity of outputs had no effect on visibility. We did not check each listed author’s name for homonymity.

**Table 2:** Left: authors with the largest number of works (${\mathbf {WA}}_D$ indeg), Right: authors with the largest contribution to the field (weighted indegree in normalized ${\mathbf {WA}}_D$)

*n*	Works	Author	Value	Author
1	61	BORNMANN_L	29.1167	BORNMANN_L
2	59	ALTMAN_D	21.7833	DANIEL_H
3	55	SMITH_R	18.2453	SMITH_R
4	55	LEE_J	18.0105	ALTMAN_D
5	50	MOHER_D	17.7255	MARSHALL_E
6	48	DANIEL_H	17.0000	GARFIELD_E
7	46	SMITH_J	15.3788	SMITH_J
8	38	CURTIS_K	15.1737	RENNIE_D
9	36	BROWN_D	14.6538	SQUIRES_B
10	36	RENNIE_D	14.5636	CHENG_J
11	35	LEE_S	13.8833	THOENNES_M
12	32	WANG_J	13.7957	COHEN_J
13	32	WILLIAMS_J	13.2898	JOHNSON_C
14	31	THOENNES_M	13.2857	REYES_H
15	29	JOHNSON_C	12.9779	LEE_J
16	29	JOHNSON_J	12.6667	WELLER_A
17	29	REYES_H	11.9167	BJORK_B
18	28	ZHANG_Y	11.1648	BROWN_D
19	28	WANG_Y	10.9091	BROWN_C
20	27	ZHANG_L	10.5000	MERVIS_J
21	27	SMITH_M	10.3762	CALLAHAM_M
22	27	WILLIAMS_A	10.2952	JONES_R
23	27	CASTAGNA_C	10.2198	MOHER_D
24	25	COHEN_J	10.0000	HARNAD_S
25	25	HELSEN_W	10.0000	BEREZIN_A

In order to calculate the author’s contribution that is shown in Table 2, we used the normalized authorship network ${\mathbf {N}} = [n_{pv}]$. A contribution of each paper *p* was equal to $\sum _v n_{pv} = 1$. Because we did not have information about each author’s real contribution, we used the so called *fractional approach* (Gauffriau et al. 2007; Batagelj and Cerinšek 2013) and set

$$\begin{aligned} n_{pv} = \frac{wa_{pv}}{{\mathrm {outdeg}}(p)}. \end{aligned}$$

This means that the contribution of an author *v* to the field is equal to its weighted indegree

$$\begin{aligned} {\text{ windeg }}(v) = \sum _{p\in W} n_{pv} \end{aligned}$$

Table 2 shows the authors who contributed more to the field of “peer review”. Comparing both panels of Table 2, it is possible to observe, for example, that L. Bornmann contributed $0.477 = 29.1167/61$ to the papers he co-authored as he collaborated with other researchers in the field. Vice-versa, for example, E. Marshall (indeg $=20$) and E. Garfield (indeg $= 17$) mostly contributed to the field as single authors and so appeared higher in the right panel of Table 2.

**Table 3:** Main authors through time

	–1970		1971–1980		1981–1990		1991–2000		2001–2005		2006–2010		2011–2015
13	CLARK_G	6	WEINSTEI_P	13	SQUIRES_B	19	RENNIE_D	13	BENNINGE_M	34	BORNMANN_L	36	LEE_J
12	FISHER_H	6	MILGROM_P	8	CHALMERS_T	16	SMITH_R	13	SMITH_R	30	DANIEL_H	31	BROWN_D
9	MILSTEAD_K	6	RATENER_P	8	COHEN_L	12	REYES_H	12	ALTMAN_D	26	ALTMAN_D	25	ZHANG_L
9	SMITH_J	6	MORRISON_K	7	CHUBIN_D	11	MARSHALL_E	12	JOHNSON_J	20	HELSEN_W	25	LEE_S
8	WILEY_F	6	ZUCKERMA_H	5	GARFIELD_E	9	LUNDBERG_G	11	CASTAGNA_C	18	ANDERSON_P	24	WANG_J
8	REINDOLL_W	5	HULKA_B	5	LOCK_S	9	KOSTOFF_R	10	RUBEN_R	17	RESNICK_D	24	CURTIS_K
8	GRIFFIN_E	5	READ_W	5	HARGENS_L	9	JOHNSON_D	10	KENNEDY_D	17	MOHER_D	23	BORNMANN_L
8	ROBERTSO_A	5	GARFIELD_E	5	RENNIE_D	8	BERO_L	9	YOUNG_E	17	KAISER_M	23	MAZEROLL_S
7	ALFEND_S	4	MERTON_R	5	MARSHALL_E	8	COHEN_J	9	WEBER_P		–	23	WANG_Y
7	SALE_J	4	WALSH_J	5	SMITH_H	8	FLETCHER_R	9	JACKLER_R	12	CURTIS_K	19	THOENNES_M
7	MARSHALL_C		–		–	8	HAYNES_R	9	JOHNS_M	11	THOENNES_M	19	WANG_H
6	HALVORSO_H	2	CHUBIN_D	3	LUNDBERG_G	8	RUBIN_H	9	SATALOFF_R	10	LEE_J	19	MOHER_D
6	CAROL_J	2	CHALMERS_T			8	FLETCHER_S	8	D’OTTAVI_S	9	CASTAGNA_C		–
	–					8	KHUDER_S	8	MOHER_D	9	SMITH_R	13	ALTMAN_D
4	GARFIELD_F						–	8	WEBER_R			13	SMITH_R
2	MERTON_R					7	ALTMAN_D		–				
						6	SQUIRES_B	5	DANIEL_H				
						5	MOHER_D	5	REYES_H				
								4	BORNMANN_L				
								4	RENNIE_D				

The first rows of Table 3 indicate the top authors in each time interval. If we restrict our attention to the authors who remained in the leading group at least for two time periods, we found a sequence starting from R. Merton (–1980) and E. Garfield (–1990), followed by D. Chubin and T. Chalmers (1971–1990), B. Squires, E. Marshall and G. Lundberg (1981–2000), and D. Rennie (1981–2005) and H. Reyes (1991–2005). D. Altman, R. Smith and D. Moher remained in the leading group for four periods (1991–2015). C. Castagna and H. Daniel were very active in the period (2001–2010). Later, the leading authors were L. Bornmann (2001–2015), M. Thoennessen, J. Lee, and K. Curtis (2006–2015).

The short names ambiguity problem started to emerge with the growth of number of different authors in the period 1991–2000 with Smith_R (R, RD, RA, RC) and Johnson_D (DM, DAW, DR, DL). In 2006–2015, we found an increasing presence of Chinese (and Korean) authors: Lee_J, Zhang_L, Lee_S, Wang_J, Wang_Y, and Wang_H. Because of the “three Zhang, four Li” effect (100 most common Chinese family names were shared by 85% of the population, Wikipedia (2016) all these names represent groups of authors. For example: Lee_J (Jaegab, Jaemu, Jae Hwa, Janette, Jeong Soon, Jin-Chuan, Ji-hoon, Jong-Kwon, Joong, Joseph, Joshua,Joy L, Ju, Juliet, etc.) and Zhang_L (L X, Lanying, Lei, Li, Lifeng, Lihui, Lin, Lina, Lixiang, Lujun).

**Table 4:** Main journals (${{\mathbf {WJ}}_D}$ indeg)

*n*	Number	Journal	*n*	Number	Journal
1	515	BMJ OPEN	21	66	ANN PHARMACOTHER
2	288	JAMA-J AM MED ASSOC	22	64	NEW ENGL J MED
3	177	PLOS ONE	23	62	CUTIS
4	175	NATURE	24	59	ANN ALLERG ASTHMA IM
5	174	SCIENTOMETRICS	25	59	BEHAV BRAIN SCI
6	174	BRIT MED J	26	59	PEDIATRICS
7	165	SCIENCE	27	57	CHEM ENG NEWS
8	127	*****	28	57	MED J AUSTRALIA
9	102	ACAD MED	29	54	J GEN INTERN MED
10	98	LANCET	30	53	MATER TODAY-PROC
11	92	SCIENTIST	31	53	J SCHOLARLY PUBL
12	91	LEARN PUBL	32	53	J NANOSCI NANOTECHNO
13	81	J AM COLL RADIOL	33	53	AM J PREV MED
14	80	PHYS TODAY	34	52	BMC PUBLIC HEALTH
15	78	ARCH PATHOL LAB MED	35	50	J SEX MED
16	78	J UROLOGY	36	50	J SPORT SCI
17	75	J ASSOC OFF AGR CHEM	37	50	MED EDUC
18	73	CAN MED ASSOC J	38	48	RES EVALUAT
19	71	ANN INTERN MED	39	48	BRIT J SPORT MED
20	67	ABSTR PAP AM CHEM S	40	47	PROCEDIA ENGINEER

**Table 5:** Main journals through time

	–1970		1971–1980		1981–1990		1991–2000
75	J ASSOC OFF AGR CHEM	24	SCIENCE	46	JAMA-J AM MED ASSOC	126	JAMA-J AM MED ASSOC
21	LANCET	20	MED J AUSTRALIA	42	SCIENCE	71	NATURE
15	BRIT MED J	18	NEW ENGL J MED	33	BEHAV BRAIN SCI	66	BRIT MED J
9	PHYS TODAY	16	AM J PSYCHIAT	32	PHYS TODAY	45	SCIENCE
7	SCIENCE	15	PHYS TODAY	29	NATURE	39	ANN INTERN MED
6	J ASSOC OFF ANA CHEM	11	JAMA-J AM MED ASSOC	27	NEW ENGL J MED	38	LANCET
4	J AM OIL CHEM SOC	10	HOSP COMMUNITY PSYCH	27	SCIENTIST	29	CAN MED ASSOC J
4	YALE LAW J	10	FED PROC	25	BRIT MED J	28	SCIENTIST
3	NATURE	10	BRIT MED J	19	CAN MED ASSOC J	26	BEHAV BRAIN SCI
3	BRIT J SURG	9	NATURE	16	PROF PSYCHOL	25	SCIENTOMETRICS
3	AM SOCIOL	9	AM SOCIOL	13	SCI TECHNOL HUM VAL	23	ACAD MED
		7	NEW YORK STATE J MED	13	S AFR MED J	23	J ECON LIT
		7	MED CARE	12	HOSPITALS		–
					–	12	PHYS TODAY
				9	LANCET	9	NEW ENGL J MED
				6	SCIENTOMETRICS		

	2001–2005		2006–2010		2011–2015		
49	JAMA-J AM MED ASSOC	44	SCIENTOMETRICS	489	BMJ OPEN		
40	CUTIS	33	JAMA-J AM MED ASSOC	146	PLOS ONE		
32	BRIT MED J	31	J SEX MED	78	SCIENTOMETRICS		
28	LEARN PUBL	27	PLOS ONE	73	J AM COLL RADIOL		
26	NATURE	27	J NANOSCI NANOTECHNO	53	MATER TODAY-PROC		
24	ABSTR PAP AM CHEM S	27	ACAD MED	47	PROCEDIA ENGINEER		
23	ACAD MED	25	SCIENTIST	47	PROCEDIA COMPUT SCI		
22	J PROSTHET DENT	25	J UROLOGY	43	ARCH PATHOL LAB MED		
22	ANN ALLERG ASTHMA IM	23	LEARN PUBL	41	BMC PUBLIC HEALTH		
18	SCIENTOMETRICS	23	J SPORT SCI	30	BMC HEALTH SERV RES		
16	J UROLOGY	23	ARCH PATHOL LAB MED	30	J ATHL TRAINING		
16	MED EDUC	21	NATURE	30	AM J PREV MED		
	–		–	29	ACAD MED		
14	LANCET	19	CUTIS		–		
13	SCIENCE	19	MED EDUC	24	LEARN PUBL		
12	SCIENTIST	19	SCIENCE	23	JAMA-J AM MED ASSOC		
		16	BRIT MED J	19	BMJ-BRIT MED J		

**Fig. 5:**
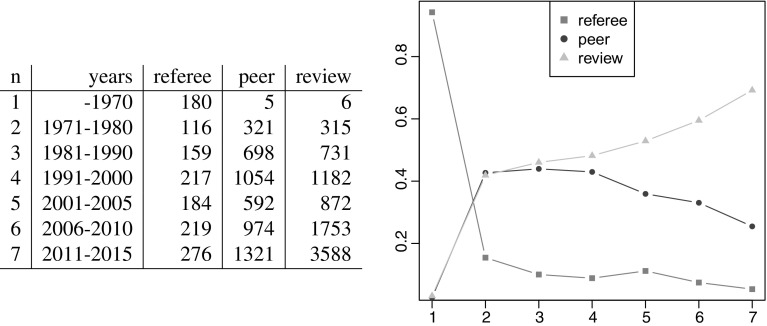
Referee: peer: review

More interestingly, our analysis showed that researchers in medicine were more active in studying peer review, though this can be simply due to the larger size of this community. Out of 47 top journals publishing papers on peer review, 23 journals were listed in medicine (see Table 4). Among these top journals, there are also *Nature*, *Science*, *Scientist*, but also specialized journals on science studies such as* Scientometrics*. The third one on the list is a rather new (from 2006) open access scientific journal, that is, *PLoS ONE*.

Table 5 indicates that the first papers on the “peer review” were published in chemistry, physics, medicine, sociology and general science journals. Some of these remained among leading journals on “peer review” also in the following periods: *Phys Today* (–2000), *Lancet* (–2005), *Science*, *Nature* (–2010), and *Brit Med J* (–2015). In the period (1971–1980) two medical journals *New Eng J Med* (1971–2000) and *JAMA* (1971–2015) joined the leading group. *JAMA* was in the period (1981–2005) the main journal. In this period, most of the leading outlets were medicine journals. In the period (1981–1990), *Scientometrics* (1981–2015) and *Scientist* (1981–2010) significantly contributed. In the period (2006–2010), *Scientometrics *was the main journal and *PLoS ONE* entered the picture of the leading group, joined in the period (2011–2015) by *BMJ Open*. Together with *Scientometrics*, these two journals were the most prolific in publishing research on peer review, whereas in the period (2011–2015), *Science*, *Nature*, *JAMA*, *BMJ *and *Learn Pub* disappeared from the top.

We also analyzed the main keywords (keywords in the papers and words in the titles). While obviously ’review’ and ’peer’ were top keywords, other more familiar in medicine appeared frequently, such as medical, health, medicine, care, patient, therapy, clinical, disease, cancer and surgery as did trial, research, quality, systematic, journal, study and analysis. More importantly, it is interesting to note that ’refereeing’ initially prevailed over ’peer review’, which became more popular later (see Fig. 5).

## Citations

**Fig. 6:**
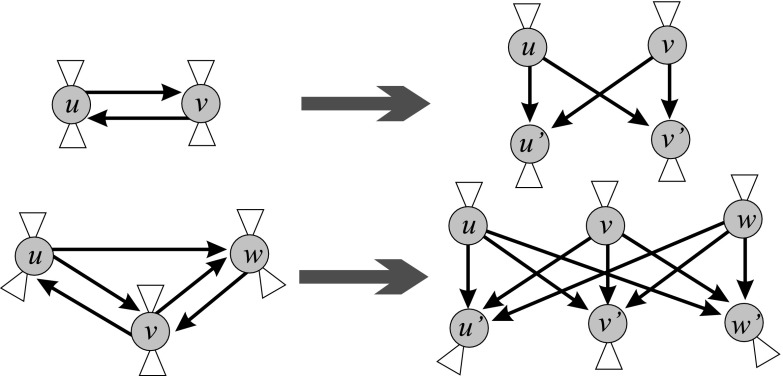
Preprint transformation

A citation network is usually (almost) acyclic. In the case of small strong components (cyclic parts) it can be transformed into a corresponding acyclic network using the *preprint transformation*. The preprint transformation replaces each work *u* from a strong component by a pair: published work *u* and its preprint version $u'$. A published work could cite only preprints. Each strong component was replaced by a corresponding complete bipartite graph on pairs—see Fig. 6 and Batagelj et al. (2014, p. 83). We determined the importance of arcs (citations) and nodes (works) using SPC (Search Path Count) weights which require an acyclic network as input data. Using SPC weights, we identified important subnetworks using different methods: main path(s), cuts and islands. Details will be given in the following subsections. Alternative approches have been proposed by Eck and Waltman (2010, 2014); Leydesdorff and Ahrweiler (2014).

We first restricted the original citation network ${\mathbf {Cite}}$ to its ‘boundary’ (45,917 nodes). This network, ${\mathbf {CiteB}}$, had one large weak component (39,533 nodes), 155 small components (the largest of sizes 191, 46, 32, 31, 18), and 5589 isolated nodes. The isolated nodes correspond to the works with WoS description, not connected to the rest of the network, and citing only works that were cited at most twice—and therefore were removed from the network ${\mathbf {CiteB}}$.

**Fig. 7:**
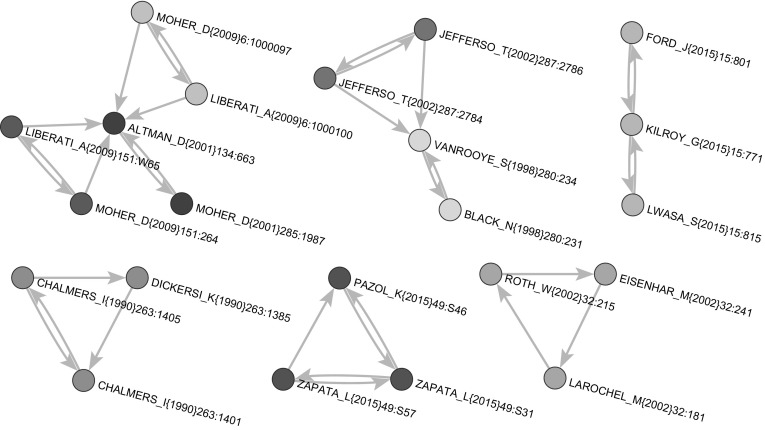
Selected strong components

**Fig. 8:**
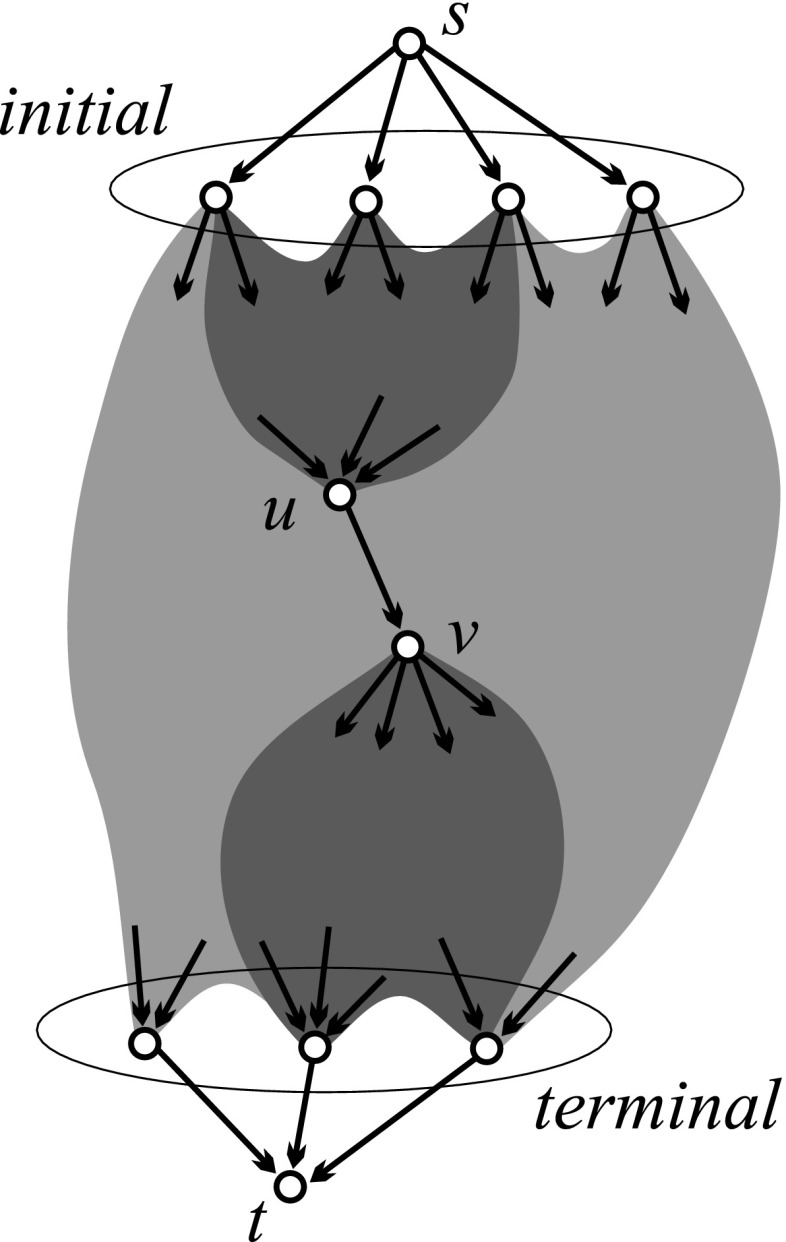
Search path count method (SPC)

The network ${{\mathbf {CiteB}}}$ includes also 22 small strong components (4 of size 3 and 18 of size 2). Figure 7 shows selected strong components. In order to apply the SPC method, we transformed the citation network in an acyclic network, ${{\mathbf {CiteAcy}}}$, using the preprint transformation. In order to make it connected, we added a common source node *s* and a common sink node *t* (see Fig. 8). The network ${{\mathbf {CiteAcy}}}$ has $n=45{,}965$ nodes and $m=132{,}601$ arcs.

### Search path count method (SPC)

The *search path count* (SPC) method (Hummon and Doreian 1989) allowed us to determine the *importance* of arcs (and also nodes) in an acyclic network based on their position. It calculates counters *n*(*u*, *v*) that count the number of different paths from some initial node (or the source *s*) to some terminal node (or the sink *t*) through the arc (*u*, *v*). It can be proved that all sums of SPC counters over a minimal arc cut-set give the same value *F*—the flow through the network. Dividing SPC counters by *F*, we obtain *normalized* SPC weights

$$\begin{aligned} w(u,v) = \frac{n(u,v)}{F} \end{aligned}$$

that can be interpreted as the probability that a random *s*-*t* path passes through the arc (*u*, *v*) (see Batagelj (2003) and Batagelj et al. (2014, pp. 75–81); this method is available in the program **Pajek**).

In the network ${\mathbf {CiteAcy}}$, the normalized SPC weights were calculated. On their basis the main path, the CPM path, main paths for 100 arcs with the largest SPC weights (“Main paths” section), and link islands [20,200] (“Cuts and islands” section) were determined.

### Main paths

In order to determine the important subnetworks based on SPC weights, Hummon and Doreian (1989) proposed the *main path method*. The *main path* starts in a link with the largest SPC weight and expands in both directions following the adjacent new link with the largest SPC weight. The *CPM path* is determined using the Critical Path Method from Operations Research (the sum of SPC weights on a path is maximal).

A problem with both main path methods is that they are unable to detect parallel developments and branchings. In July 2015 a new option was added to the program **Pajek**:

$$\begin{aligned} {\texttt {Network/acyclic network/create (sub)network/main paths}} \end{aligned}$$

with several suboptions for computing local and global main paths and for searching for Key-Route main path in acyclic networks (Liu and Lu 2012). Here, the procedure begins with a set of selected *seed arcs* and expands them in both directions as in the main path procedure.

**Fig. 9:**
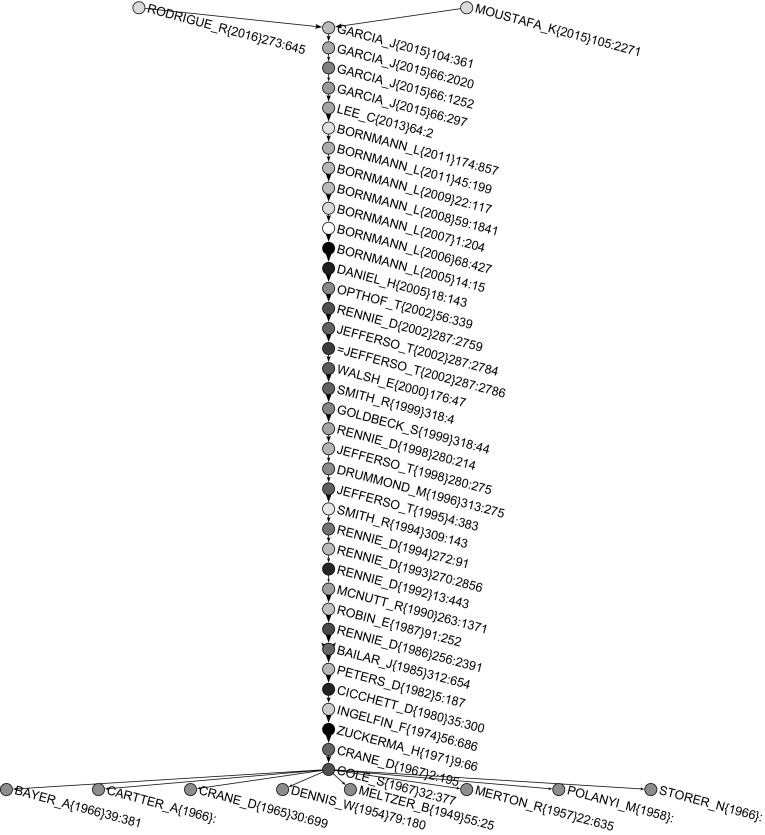
Main path

**Fig. 10:**
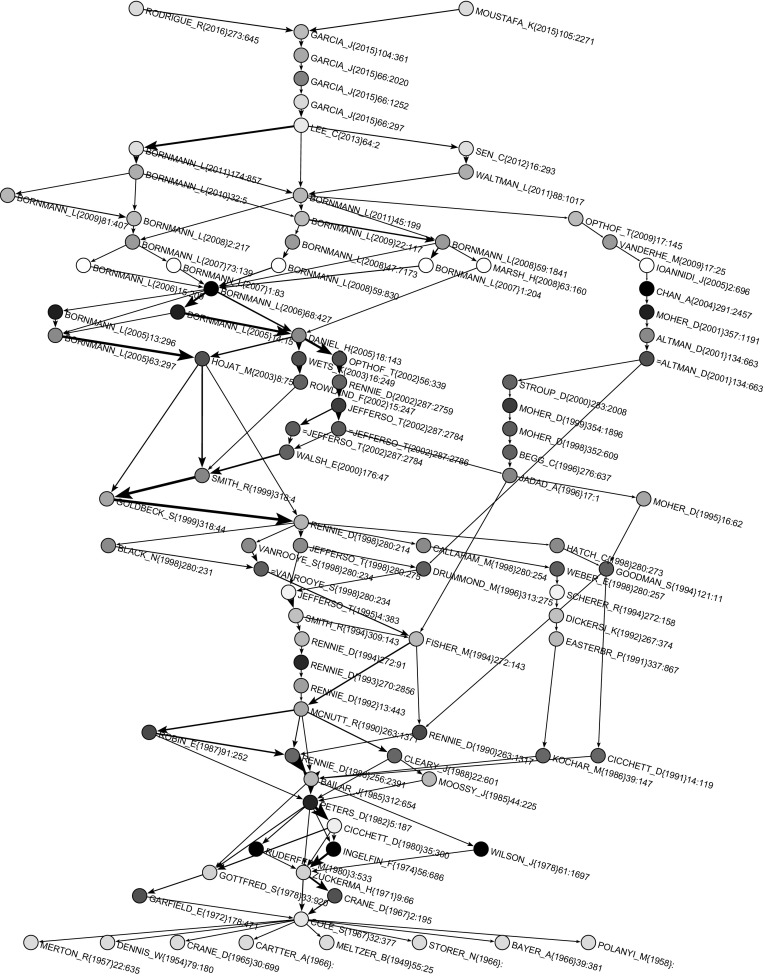
Main paths for 100 largest weights

Both main path and CPM procedure gave the same main path network presented in Fig. 9. Nodes with a name starting with = (for axample =JEFFERSO_T(2002)287-2786 in Fig. 9) correspond to a preprint version of a paper. In Fig. 10, main paths for 100 seed arcs with the largest SPC weights are presented. The main path was included in this subnetwork and there were additional 47 works on parallel paths. Many of these additional works were from authors of the main path (e.g., Rennie, Cicchetti, Altman, Bornmann, Opthof). It is interesting that Moher’s publications appear on main paths four times. He is also among the most cited authors and among authors who had the highest number of publications, but he did not appear on the main path.

### Main path publication pattern

Our analysis found 48 works on the main path. After looking at all these works in detail, we classified them into three groups determined by their time periods:

- Before 1982: this includes works published mostly in social science and philosophy journals and social science books;
- From 1983 to 2002: this includes works published almost exclusively in biomedical journals;
- From 2003: this includes works published in specialized science studies journals.

#### The main path till 1982

This period includes important social science journals, such as *American Journal of Sociology*, *American Sociologist*, *American Psychologist* and *Sociology of Education*, and three foundational books. The most influential authors were: Meltzer (1949), Dennis (1954), Merton (1957), Polany (1958), Crane (1965, 1967), Bayer and Folger (1966), Storer (1966), Cartter (1966), Cole and Cole (1967), Zuckerman and Merton (1971), Ingelfinger (1974), Cicchetti (1980), and Peters and Ceci (1982). The most popular topics were: scientific productivity, bibliographies, knowledge, citation measures as measures of scientific accomplishment, scientific output and recognition, evaluation in science, referee system, journal evaluation, peer-evaluation system, review process, peer review practices.

#### The main path from 1983 to 2002

This period includes biomedical journals, mainly *JAMA*. It is worth noting that *JAMA* published many papers which were presented at the *International Congress on Peer Review and Biomedical Publication* since 1986. Among the more influential authors were: Rennie (1986, 1992, 1993, 1994, 2002), Smith (1994, 1999), and Jefferson with his collaborators Demicheli, Drummond, Smith, Yee, Pratt, Gale, Alderson, Wager and Davidoff (1995, 1998, 2002). The most popular topics were: the effects of blinding on review quality, research into peer review, guidelines for peer reviewing, monitoring the peer review performance, open peer review, bias in peer review system, measuring the quality of editorial peer review, development of meta-analysis and systematic reviews approaches.

#### The main path from 2003

Here, the situation changed again. Some specialized journals on science studies gained momentum, such as *Scientometrics*, *Research Evaluation*, *Journal of Informetrics* and* JASIST*. The most influential authors were: Bornmann and Daniel (2005, 2006, 2007, 2008, 2009, 2011) and Garcia, Rodriguez-Sanchez and Fdez-Valdivia (4 papers in 2015, 2016). Others popular publications were Lee et al. (2013) and Moustafa (2015). Research interest went to peer review of grant proposals, bias, referee selection and editor-referee/author links.

### Cuts and islands

Cuts and islands are two approaches to identify important groups in a network. The importance is expressed by a selected property of nodes or links.

**Fig. 11:**
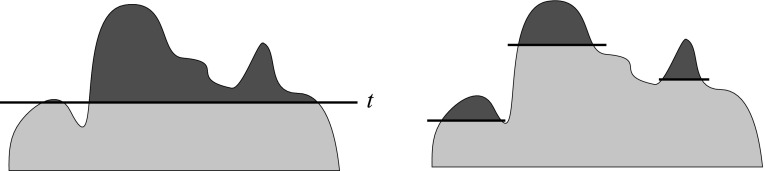
Cuts and islands

If we represent a given or computed property of nodes/links as a height of nodes/links and we immerse the network into a water up to a selected property threshold level, we obtain a cut (see the left picture in Fig. 11). By varying the level, we can obtain different *islands*—maximal connected subnetwork such that values of selected property inside island are larger than values on the island’s neighbors and the size (number of island’s nodes) is within a given range [*k*, *K*] (see the right picture in Fig. 11). An island is *simple* iff it has a single peak [for details, see (Batagelj et al. 2014, pp. 54–61)].

Zaveršnik and Batagelj (2004) developed very efficient algorithms to determine the islands hierarchy and list all the islands of selected sizes. They are available in **Pajek**.

Islands allow us also to overcome a typical problem of the main path approach, that is the selection of seed arcs. Here, we simply determined all islands and looked at the maximal SPC weight in each island. This allowed us to determine the importance of an island.

**Fig. 12:**
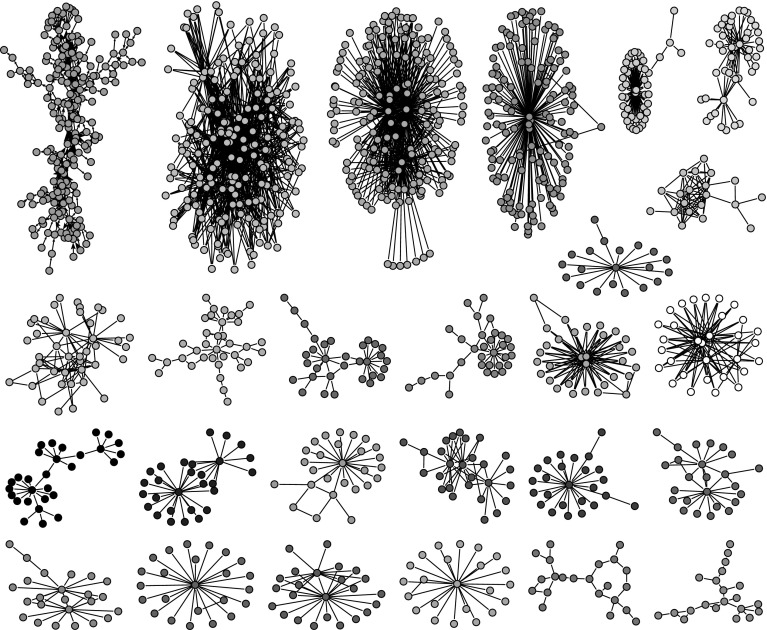
SPC islands [20,200]

When searching for SPC link islands for the number of nodes between 20 and 200 (and between 20 and 100), we found 26 link islands (see Fig. 12). Many of these islands have a very short longest path, often a star-like structure (a node with its neighbors). These islands are not very interesting for our purpose. We visually identified “interesting” islands and inspected them in detail. In the following list, we present basic information for each of selected island, i.e., the number of nodes for the selection of 20–200 nodes (and 20–100), the maximal SPC weight in the island and a short description of the island:

- Island 1. $n = 191 (99)$, 0.297. Peer-review.
- Island 2. $n = 191 (96)$, $0.211 \times 10^{-8}$. Discovery of different isotopes.
- Island 3. $$n = 178$$, $$0.165 \times 10^{-8}$$. Biomass.
- Island 7. $n = 42$, $0.425 \times 10^{-8}$. Athletic trainers.
- Island 8. $n = 36$, $0.191 \times 10^{-4}$ Sport refereeing and decision-making.
- Island 9. $n = 32$, $0.793 \times 10^{-10}$. Environment pollution.
- Island 13. $n = 29$, $0.451 \times 10^{-10}$. Toxicity testing.
- Island 23. $n = 22$, $0.344 \times 10^{-8}$. Peer-review in psychological sciences.
- Island 24. $n = 21$, $0.487 \times 10^{-10}$. Molecular interaction.

Only Island 1 and Island 23 dealt directly with the peer review. Other islands represented collateral stories. The Island 1 on peer-review was the most important because it had the maximal SPC weight at least 10.000 times higher than the next one, i.e., Island 8 on sport refereeing.

**Fig. 13:**
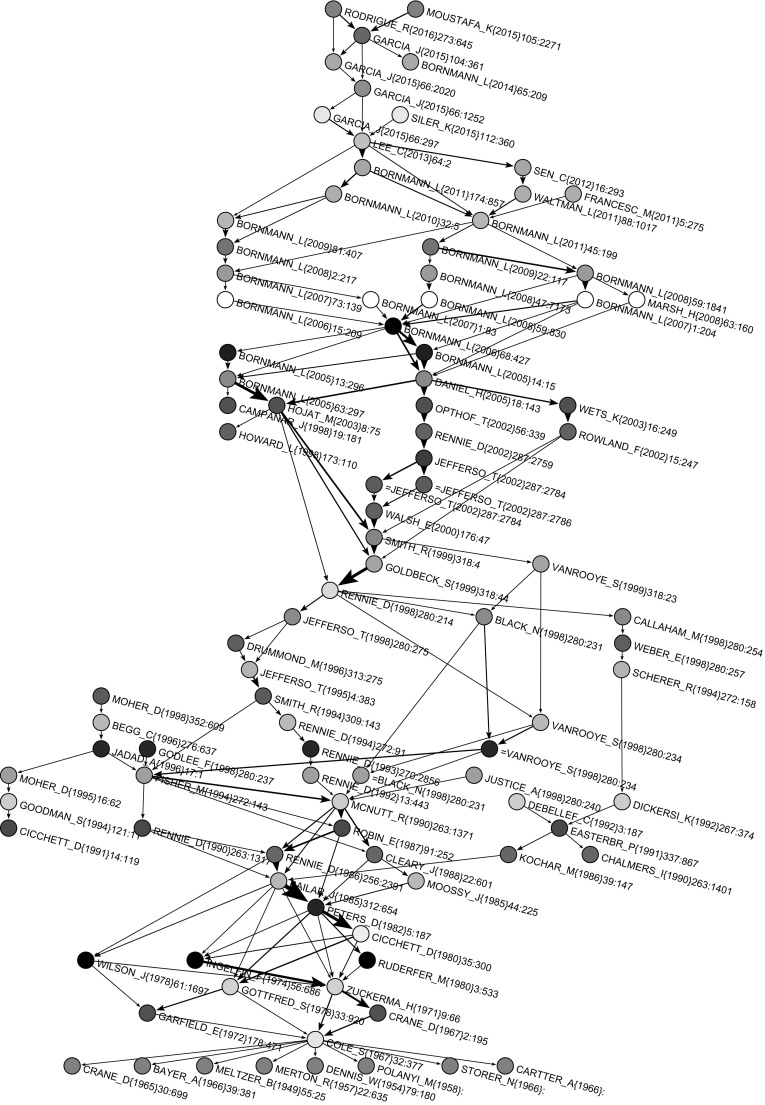
SPC link Island 1 [100]

For the sake of readability, we extracted from Island 1 a sub-island of size in range [20, 100], which is shown in Fig. 13. It contains the main path and strongly overlaps with the main paths in Fig. 8. The list of all publications from the main path (coded with 1), main paths (coded with 2) and SPC link island (20–100) (coded with 3) is shown in Table 6 in the “Appendix”. We found 105 works in the joint list. Only 9 publications were exclusively on main paths and only 10 publications were exclusively in the SPC link island. The three groups typology of works also held for the list of all 105 publications.

## Conclusions

This article provided a quantitative analysis of peer review as an emerging field of research by revealing patterns and connections between authors, fields and journals from 1950 to 2016. By collecting all available sources from WoS, we were capable of tracing the emergence and evolution of this field of research by identifying relevant authors, publications and journals, and revealing important development stages. By constructing several one-mode networks (i.e., co-authorship network, citation network) and two-mode networks, we found connections and collective patterns.

However, our work has certain limitations. First, given that data were extracted from WoS, works from disciplines and journals less covered by this tool could have been under-represented. This especially holds for humanities and social sciences, which are less comprehensively covered by WoS and more represented in Scopus and even more in GoogleScholar (e.g., Halevi et al. 2017), which also lists books and book chapters (e.g., Halevi et al. 2016). However, given that GoogleScholar does not permit large-scale data collection, a possible validation of our findings by using Scopus could be more feasible.

Furthermore, given that data were obtained using the queries “peer review*” and refereeing and that these terms could be used in many fields, e.g., sports, our dataset included some works that probably had little to do with peer review as a research field. For example, when reading the abstracts of certain works included in our dataset, we found works reporting ’Published by Elsevier Ltd. Selection and/or peer review under responsibility of’. An extra effort (unfortunately almost prohibitive) in cleaning the dataset manually would help filtering out irrelevant records. However, by using the main path and island methods, we successfully identified the most important and relevant publications on peer review without incurring in excessive cost of data cleaning or biasing our findings significantly.

Secondly, another limitation of our work is that we did not treat author name disambiguation, as evident in Table 3. This could be at least partially solved by developing automatic disambiguation procedures, although the right solution would be the adoption by WoS and publishers of the standards such as ResearcherID and ORCID to allow for a clear identification since from the beginning. To control for this, we could include in **WoS2Pajek** additional options to create short author names that will allow manual correction of names of critical authors.

With all these caveats, our study allowed us to circumscribe the field, capture its emergence and evolution and identify the most influential publications. Our main path procedures and islands method used SPC weights on citation arcs. It is important to note that the 47 publications from the main path were found in all other obtained lists of the most influential publications. They could be considered as the main corpus of knowledge for any newcomer in the field. More importantly, at least to have a dynamic picture of the field, we found these publications to be segmented in three phases defined by specific three time periods: before 1982, with works mostly published in social sciences journals (sociology, psychology and education); from 1983 to 2002, with works published almost exclusively in biomedical journals, mainly *JAMA*; and after 2003, with works published more preferably in science studies journals (e.g., *Scientometrics*, *Research Evaluation*, *Journal of Informetrics*).

This typology indicates the emergence and evolution of peer review as a research field. Initiatives to promote data sharing on peer review in scholarly journals and funding agencies (e.g., Casnici et al. 2017; Squazzoni et al. 2017) as well as the establishment of regular funding schemes to support research on peer review would help to strengthen the field and promote tighter connections between specialists.

Results also showed that while the term “peer review” itself was relatively unknown before 1970 (“referee” was more frequently used), publications on peer review significantly grew especially after 1990.

## Appendix

### The structure of names in constructed networks

The usual *ISI name* of a work as used in the CR field, e.g.,  has the following structure  where AU$_{1}$ is the first author’s name and SO[:20] is the string of the initial (up to) 20 characters in the SO field.

In WoS records, the same work can have different ISI names. To improve precision, the program **WoS2Pajek** supports also *short names* [similar to the names used in HISTCITE output (Garfield et al. 2003)]. They have the format:

For example: TREGENZA_T(2002)17:349. From the last names with prefixes VAN, DE, etc. the space is deleted. Unusual names start with a character * or $. The name [ANONYMOUS] is used for anonymous authors.

This construction of names of works provides a good balance between the synonymy problem (different names designating the same work) and the homonymy problem (a name designating different works). We treated the remaining synomyms and homonyms in the network data as a noise. If their effect surfaces into final results, we either corrected our copy of WoS data and repeated the analysis, or, if the correction required excessive work, simply reported the problem. A typical such case was the author name [ANONYMOUS] or combinations with some very frequent last names—in MathSciNet there are 85 mathematicians corresponding to the short name SMITH_R and 1792 mathematicians corresponding to the short name WANG_Y.

The composed keywords were decomposed in single words. For example, ‘peer review’ into ‘peer’ and ‘review’. On keywords obtained from titles of works we applied the lemmatization (using the Monty Lingua library).

The name ***** denoted a missing journal name.

### Details about important works

In Tables 6, 7 and 8 a list of works on main path (1), main paths (2) and island (3) is presented. Only the first authors are listed.

**Table 6:** List of works on main path (1), main paths (2) and island (3)—part 1

Year	Code	First author	Title	Journal
1949	123	Meltzer, BN	The productivity of social scientists	AM J SOCIOL
1954	123	Dennis, W	Bibliographies of eminent scientists	SCIENTIFIC M
1957	123	Merton, RK	Priorities in scientific discovery—a chapter in the sociology of science	AM SOCIOL REV
1958	123	Polanyi, M	Personal knowledge: towards a post-critical philosophy	UP Chicago
1965	123	Crane, D	Scientists at major and minor universities	AM SOCIOL REV
1966	123	Bayer, AE	Some correlates of citation measure of productivity in science	SOCIOL EDUC
1966	123	Storer, NW	The social system of science	HRW
1966	123	Cartter, A	An Assessment of quality in graduate education	ACE
1967	123	Crane, D	Gatekeepers of science—some factors affecting selection of articles...	AM SOCIOL
1967	123	Cole, S	Scientific output and recognition—study in operation of reward system...	AM SOCIOL REV
1971	123	Zuckerma.H	Patterns of evaluation in science—...of referee system	MINERVA
1972	23	Garfield, E	Citation analysis as a tool in journal evaluation—journals can be ranked...	SCIENCE
1974	123	Ingelfin.FJ	Peer review in biomedical publication	AM J MED
1978	23	Wilson, JD	70th annual-meeting of american-society-for-clinical-investigation,...	J CLIN INVEST
1978	23	Gottfredson, SD	Evaluating psychological-research reports—...of quality judgments	AM PSYCHOL
1980	23	Ruderfer, M	The fallacy of peer-review—judgment without science and a case-history	SPECULAT SCI TECHNOL
1980	123	Cicchetti, DV	Reliability of reviews for the american-psychologist...	AM PSYCHOL
1982	123	Peters, DP	Peer-review practices of psychological journals—the fate...	BEHAV BRAIN SCI
1985	123	Bailar, JC	Journal peer-review—the need for a research agenda	NEW ENGL J MED
1985	23	Moossy, J	Anonymous authors, anonymous referees—an editorial exploration	J NEUROPATH EXP NEUR
1986	123	Rennie, D	Guarding the guardians—a conference on editorial peer-review	JAMA
1986	23	Kochar, MS	The peer-review of manuscripts in need for improvement	J CHRON DIS
1987	123	Robin, ED	Peer-review in medical journals	CHEST
1988	23	Cleary, JD	Blind versus nonblind review—survey of selected medical journals	DRUG INTEL CLIN PHAR
1990	123	Mcnutt, RA	The effects of blinding on the quality of peer-review—a randomized trial	JAMA
1990	23	Rennie, D	Editorial peer-review in biomedical publication—the 1st-international-congress	JAMA
1990	3	Chalmers, I	A cohort study of summary reports of controlled trials	JAMA
1991	23	Cicchetti, DV	The reliability of peer-review for manuscript and grant submissions...	BEHAV BRAIN SCI
1991	23	Easterbrook, PJ	Publication bias in clinical research	LANCET
1992	3	Debellefeuille, C	The fate of abstracts submitted to a cancer meeting...	ANN ONCOL
1992	123	Rennie, D	Suspended judgment—editorial peer-review—let us put it on trial	CONTROL CLIN TRIALS
1992	23	Dickersin, K	Factors influencing publication of research results—follow-up of...	JAMA
1993	123	Rennie, D	More peering into editorial peer-review	JAMA
1994	23	Scherer, RW	Full publication of results initially presented in abstracts—a metaanalysis	JAMA
1994	23	Goodman, SN	Manuscript quality before and after peer-review and editing at Annals...	ANN INTERN MED

**Table 7:** List of works on main path (1), main paths (2) and island (3)—part 2

Year	Code	First author	Title	Journal
1994	23	Fisher, M	The effects of blinding on acceptance of research papers by peer-review	JAMA
1994	123	Rennie, D	The 2nd international-congress on peer-review in biomedical publication	JAMA
1994	123	Smith, R	Promoting research into peer-review	BRIT MED J
1995	123	Jefferson, T	Are guidelines for peer-reviewing economic evaluations necessary...	HEALTH ECON
1995	23	Moher, D	Assessing the quality of randomized controlled trials...	CONTROL CLIN TRIALS
1996	23	Jadad, AR	Assessing the quality of reports of randomized clinical trials...	CONTROL CLIN TRIALS
1996	123	Drummond, MF	Guidelines for authors and peer reviewers of economic submissions to the BMJ	BRIT MED J
1996	23	Begg, C	Improving the quality of reporting of randomized controlled trials—the CONSORT statement	JAMA
1998	3	Godlee, F	Effect on the quality of peer review of blinding reviewers and...	JAMA
1998	3	Justice, AC	Does masking author identity improve peer review quality?—a randomized controlled trial	JAMA
1998	23	Weber, EJ	Unpublished research from a medical specialty meeting—Why investigators fail to publish	JAMA
1998	23	van Rooyen, S	Effect of blinding and unmasking on the quality of peer review—A randomized trial	JAMA
1998	23	Black, N	What makes a good reviewer and a good review for a general medical journal?	JAMA
1998	3	Campanario, JM	Peer review for journals as it stands today—Part 1	SCI COMMUN
1998	123	Jefferson, T	Evaluating the BMJ guidelines for economic submissions...	JAMA
1998	3	Howard, L	Peer review and editorial decision-making	BRIT J PSYCHIAT
1998	123	Rennie, D	Peer review in Prague	JAMA
1998	2	Hatch, CL	Perceived value of providing peer reviewers with abstracts and preprints...	JAMA
1998	23	Moher, D	Does quality of reports of randomised trials affect estimates of intervention efficacy...	LANCET
1998	23	Callaham, ML	Positive-outcome bias and other limitations in the outcome of research abstracts...	JAMA
1999	3	van Rooyen, S	Effect of open peer review on quality of reviews and on reviewers’ recommendations...	BRIT MED J
1999	123	Smith, R	Opening up BMJ peer review—a beginning that should lead to complete transparency	BRIT MED J
1999	123	Goldbeck-Wood, S	Evidence on peer review—scientific quality control or smokescreen?	BRIT MED J
1999	2	Moher, D	Improving the quality of reports of meta-analyses of randomised controlled trials: QUOROM	LANCET
2000	123	Walsh, E	Open peer review: a randomised controlled trial	BRIT J PSYCHIAT
2000	2	Stroup, DF	Meta-analysis of observational studies in epidemiology—A proposal for reporting	JAMA
2001	2	Altman, DG	The revised CONSORT statement for reporting randomized trials...	ANN INTERN MED
2001	2	Moher, D	The CONSORT statement: revised recommendations for improving the quality of reports...	LANCET
2002	123	Jefferson, T	Effects of editorial peer review—a systematic review	JAMA
2002	123	Jefferson, T	Measuring the quality of editorial peer review	JAMA
2002	123	Rennie, D	Fourth international congress on peer review in biomedical publication	JAMA
2002	23	Rowland, F	The peer-review process	LEARN PUBL
2002	123	Opthof, T	The significance of the peer review process against the background of bias...	CARDIOVASC RES
2003	23	Hojat, M	Impartial judgment by the “gatekeepers” of science:...	ADV HEALTH SCI EDUC
2003	23	Wets, K	Post-publication filtering and evaluation: Faculty of 1000	LEARN PUBL

**Table 8:** List of works on main path (1), main paths (2) and island (3)—part 3

Year	Code	First author	Title	Journal
2004	2	Chan, AW	Empirical evidence for selective reporting of outcomes in randomized trials...	JAMA
2005	23	Bornmann, L	Selection of research fellowship recipients by committee peer review...	SCIENTOMETRICS
2005	123	Daniel, HD	Publications as a measure of scientific advancement and of scientists’ productivity	LEARN PUBL
2005	123	Bornmann, L	Committee peer review at an international research foundation:...	RES EVALUAT
2005	23	Bornmann, L	Criteria used by a peer review committee for selection of research fellows...	INT J SELECT ASSESS
2005	2	Ioannidis, JPA	Why most published research findings are false	PLOS MED
2006	123	Bornmann, L	Selecting scientific excellence through committee peer review—a citation analysis...	SCIENTOMETRICS
2006	23	Bornmann, L	Potential sources of bias in research fellowship assessments:...	RES EVALUAT
2007	123	Bornmann, L	Convergent validation of peer review decisions using the h index...	J INFORMETR
2007	23	Bornmann, L	Gatekeepers of science—effects of external reviewers’ attributes...	J INFORMETR
2007	23	Bornmann, L	Row-column (RC) association model applied to grant peer review	SCIENTOMETRICS
2008	23	Marsh, HW	Improving the peer-review process for grant applications...	AM PSYCHOL
2008	123	Bornmann, L	Selecting manuscripts for a high-impact journal through peer review...	J AM SOC INF SCI TEC
2008	23	Bornmann, L	The effectiveness of the peer review process: inter-referee agreement...	ANGEW CHEM INT EDIT
2008	23	Bornmann, L	Latent Markov modeling applied to grant peer review	J INFORMETR
2008	23	Bornmann, L	Are there better indices for evaluation purposes than the h index?...	J AM SOC INF SCI TEC
2009	123	Bornmann, L	The luck of the referee draw: the effect of exchanging reviews	LEARN PUBL
2009	2	Opthof, T	The Hirsch-index: a simple, new tool for the assessment of scientific output...	NETH HEART J
2009	2	van der Heyden, MAG	Fraud and misconduct in science: the stem cell seduction	NETH HEART J
2009	23	Bornmann, L	The influence of the applicants’ gender on the modeling of a peer review...	SCIENTOMETRICS
2010	23	Bornmann, L	The manuscript reviewing process: Empirical research on review...	LIBR INFORM SCI RES
2011	123	Bornmann, L	Scientific Peer Review	ANNU REV INFORM SCI
2011	123	Bornmann, L	A multilevel modelling approach to investigating the...of editorial decisions:...	J R STAT SOC A STAT
2011	3	Franceschet, M	The first Italian research assessment exercise: A bibliometric perspective	J INFORMETR
2011	23	Waltman, L	On the correlation between bibliometric indicators and peer review:...	SCIENTOMETRICS
2012	23	Sen, CK	Rebound peer review: a viable recourse for aggrieved authors?	ANTIOXID REDOX SIGN
2013	123	Lee, CJ	Bias in peer review	J AM SOC INF SCI TEC
2014	3	Bornmann, L	Do we still need peer review? an argument for change	J ASSOC INF SCI TECH
2015	3	Siler, K	Measuring the effectiveness of scientific gatekeeping	P NATL ACAD SCI USA
2015	123	Garcia, JA	The principal-agent problem in peer review	J ASSOC INF SCI TECH
2015	123	Garcia, JA	Adverse selection of reviewers	J ASSOC INF SCI TECH
2015	123	Moustafa, K	Don’t infer anything from unavailable data	SCIENTOMETRICS
2015	123	Garcia, JA	Bias and effort in peer review	J ASSOC INF SCI TECH
2015	123	Garcia, JA	The author–editor game	SCIENTOMETRICS
2016	123	Rodriguez-Sanchez, R	Evolutionary games between authors and their editors	APPL MATH COMPUT
